# A Primary Parotid Mucosa-Associated Lymphoid Tissue Non-Hodgkin Lymphoma in a Patient With Sjogren Syndrome

**DOI:** 10.7759/cureus.15679

**Published:** 2021-06-16

**Authors:** Michael R Povlow, Mitchell Streiff, Sunthosh Madireddi, Couger Jaramillo

**Affiliations:** 1 Department of Radiology, Brooke Army Medical Center, San Antonio, USA; 2 Department of Radiology, Ponce Health Sciences University, Ponce, USA; 3 Department of Pathology, Brooke Army Medical Center, San Antonio, USA

**Keywords:** parotid tumor, non-hodgkin’s lymphomas, salivary gland neoplasm, mucosa-associated lymphoid tissue (malt), head and neck neoplasms, head and neck radiology, sjogren's

## Abstract

The salivary gland tumors are rare entities and the majority of these are benign. However, there are some entities such as prior neck radiation, certain infections, and systemic diseases which should raise the clinical suspicion for a malignant lesion. Patients with Sjogren syndrome are at increased risk for a salivary gland neoplasm, specifically non-Hodgkin lymphoma. While clinical findings play an important role in the initial workup, imaging plays a critical role in the diagnosis and management. This case describes a patient with Sjogren syndrome who presented with a left face mass where imaging was able to confidently diagnose her with a suspicious parotid neoplasm with lymphoma as the favored diagnosis. After histological evaluation, she was diagnosed with primary parotid mucosa-associated lymphoid tissue (MALT) non-Hodgkin lymphoma after which she went on to non-operative management.

## Introduction

The salivary gland tumors are rare, accounting for only 6-8% of all head and neck tumors annually in the United States [1]. The risk factors including head and neck radiation [2,3], smoking, and certain viral infections, such as Epstein-Barr virus (EBV), HIV, and human papillomavirus (HPV), increase the risk for the development of these neoplasms [1,4,5]. Benign parotid gland neoplasms constitute the vast majority of all salivary gland tumors. The histological examination of these neoplasms most often reveals a pleomorphic adenoma, Warthin adenoma, basal cell adenoma, or canalicular adenoma. Approximately one-quarter of parotid gland neoplasms are malignant, with mucoepidermoid carcinoma or adenoid cystic carcinoma as the most common subtypes [1-3]. A very small subset, 2%, of malignant parotid gland neoplasms is of the non-Hodgkin lymphoma type [6]. Here, we present the case of a patient with Sjogren syndrome who developed a primary mucosa-associated lymphoid tissue (MALT) non-Hodgkin lymphoma of the parotid gland. Despite being exceedingly rare when considering parotid gland neoplasms collectively, previous case studies have suggested a strong correlation between MALT lymphoma of the parotid gland in the setting of Sjogren syndrome. Sjogren syndrome is an autoimmune disease that attacks the exocrine glands, especially the salivary glands, and leads to a significant B cell proliferation [7]. This expansive proliferation of B cells is a risk factor for the development of salivary gland lymphoma, and previous studies have concluded that the risk may be as much as seven to 19 times greater in a patient with Sjogren syndrome [8]. When considered the background of Sjogren syndrome, MALT lymphoma of the parotid gland needs to be considered on the differential diagnosis of a patient presenting with signs and symptoms of a parotid gland neoplasm. Imaging plays a crucial role in the diagnosis of salivary gland tumors. This case demonstrates the importance of early imaging and how certain imaging findings can help differentiate these various tumors to ensure the best clinical outcome for the patient.

## Case presentation

**Figure 1:**
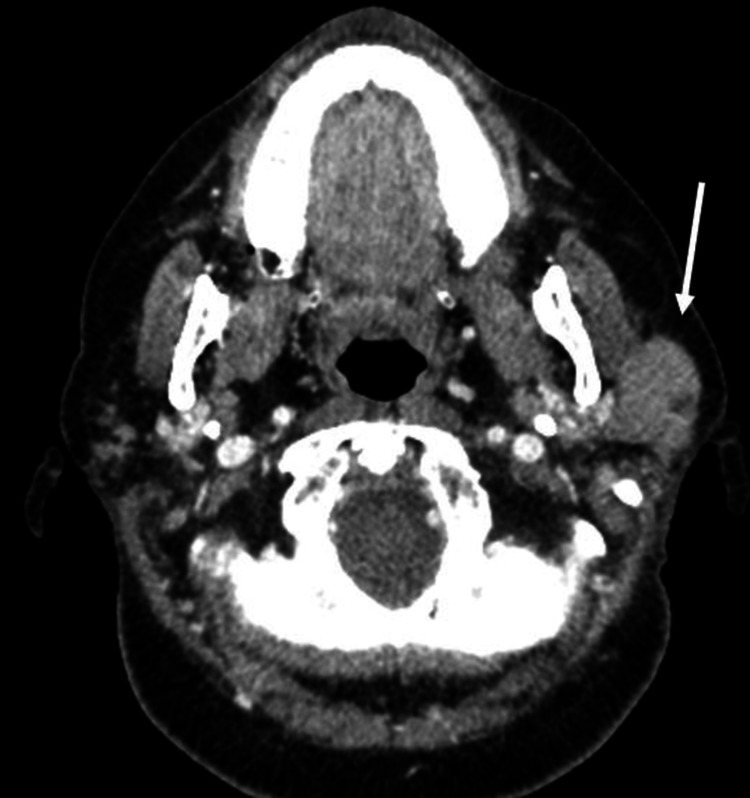
Axial CT image of the neck with intravenous contrast at the level of the parotid glands showing asymmetric left parotid gland enlargement with replacement by a soft tissue mass (white arrow).

A 57-year-old female was presented to the ED with swelling on the left face for eight months as well as unintentional 10 lb weight loss over the same time. Additional symptoms included frequent bouts of acute nervousness and tinnitus. She denied fevers and night sweats and had no pain or erythema associated with the swelling. Initial laboratory results demonstrated normal white blood cell and platelet count. The patient's kidney function and red blood cell count were normal. CT of the neck with intravenous contrast performed in the emergency department showed asymmetric enlargement of the left parotid gland (Figure 1). Of note, her submandibular glands were atrophic. The report recommended further evaluation with MRI.

**Figure 2:**
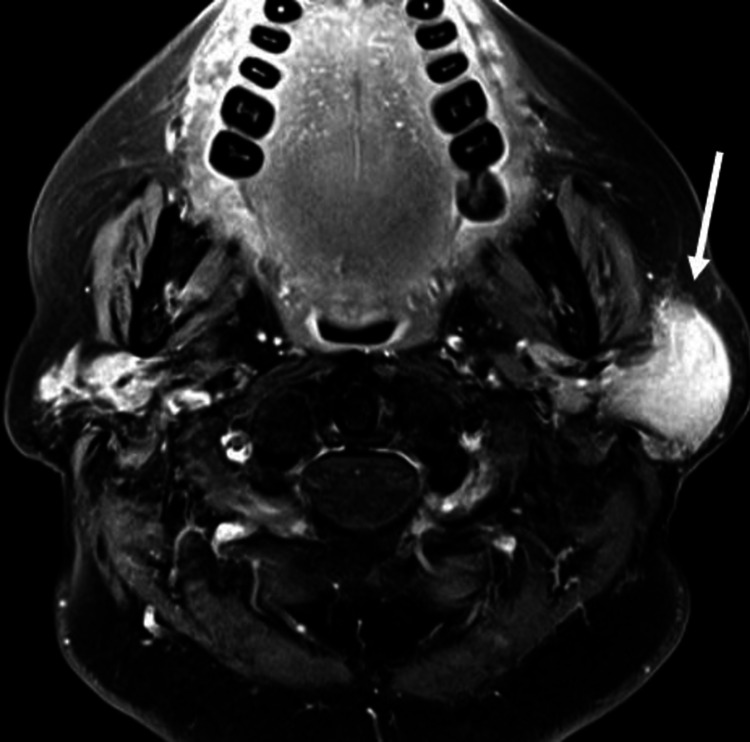
Axial fat-saturated post-contrast MRI at the level of the parotid glands showing homogeneous enhancement of left parotid space mass (white arrow). The irregular margins show infiltration into the deep parotid space at its medial aspect.

**Figure 3:**
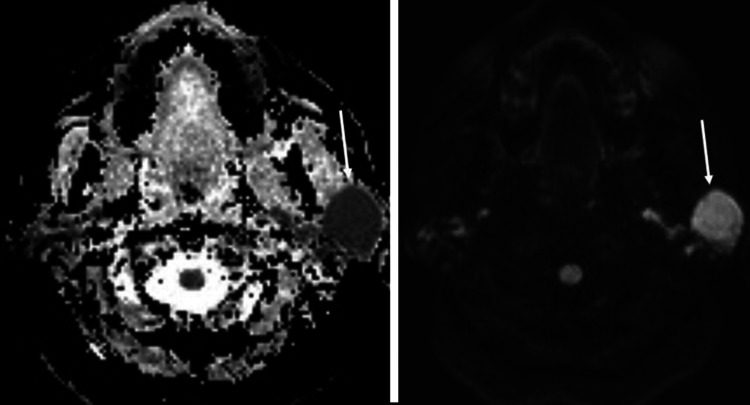
Axial ADC, left, and axial DWI, right, both at the level of the parotids. The ADC map shows a very low signal with a corresponding high signal on DWI of the left parotid mass (white arrows). This is consistent with significantly restricted diffusion. ADC: apparent diffusion coefficient; DWI: diffusion-weighted imaging

The patient was discharged and she saw her primary care provider one week later. An MRI of the face without and with intravenous contrast was ordered and performed two weeks later. It was at this time where her history of Sjogren syndrome was described in the medical record. The MRI revealed a left parotid space mass with ill-defined borders which had decreased T1 and increased T2 signal and homogeneous contrast enhancement with ill-defined margins (Figure 2). Diffusion-weighted imaging (DWI) demonstrated significantly restricted diffusion with very low apparent diffusion coefficient (ADC) values (Figure 3). The right parotid gland as well as both submandibular glands were atrophic. These findings were highly suggestive of a malignant parotid neoplasm, with the favored diagnosis of primary parotid lymphoma.

**Figure 4:**
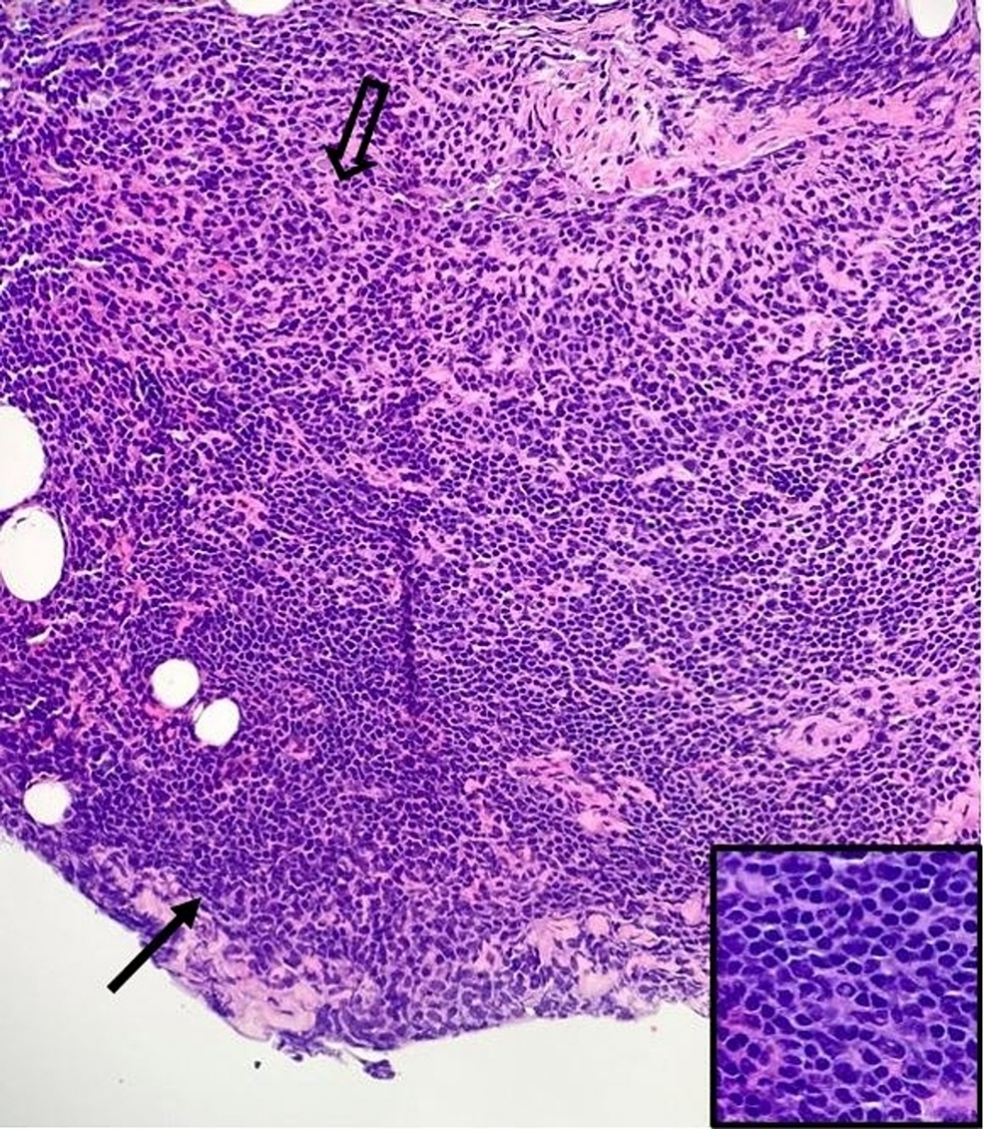
Hematoxylin and eosin stain (40x objective lens) showing extra-nodal marginal zone (MALT) lymphoma characterized by diffuse infiltration by atypical lymphocytes including small cells with irregular nuclei with inconspicuous nucleoli (black solid arrow) and larger cells with relatively abundant cytoplasm (open black arrow). Inset (bottom right, 100x oil immersion lens) demonstrates rare Dutcher bodies. MALT: mucosa-associated lymphoid tissue

**Figure 5:**
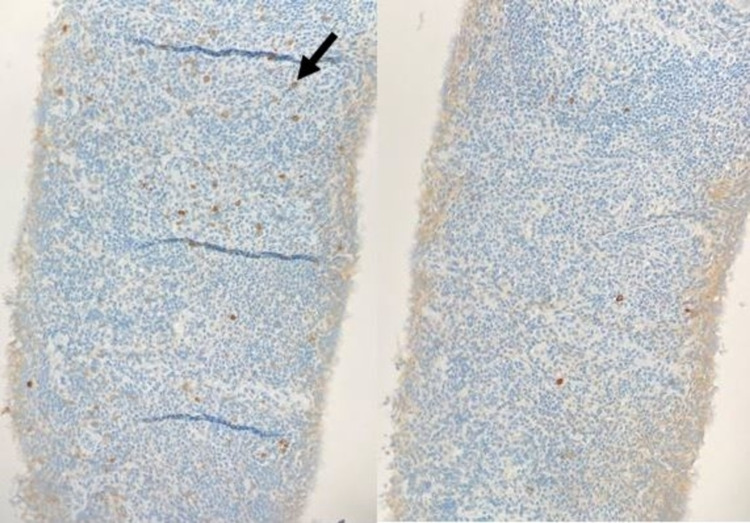
Immunohistochemistry (20x objective lens) for kappa (left) and lambda (right) with brown-stained cells consistent with positive stain uptake. There is a small population of kappa-restricted cells (black arrow on example positive cell on left). No significant uptake of lambda stain on the right.

**Figure 6:**
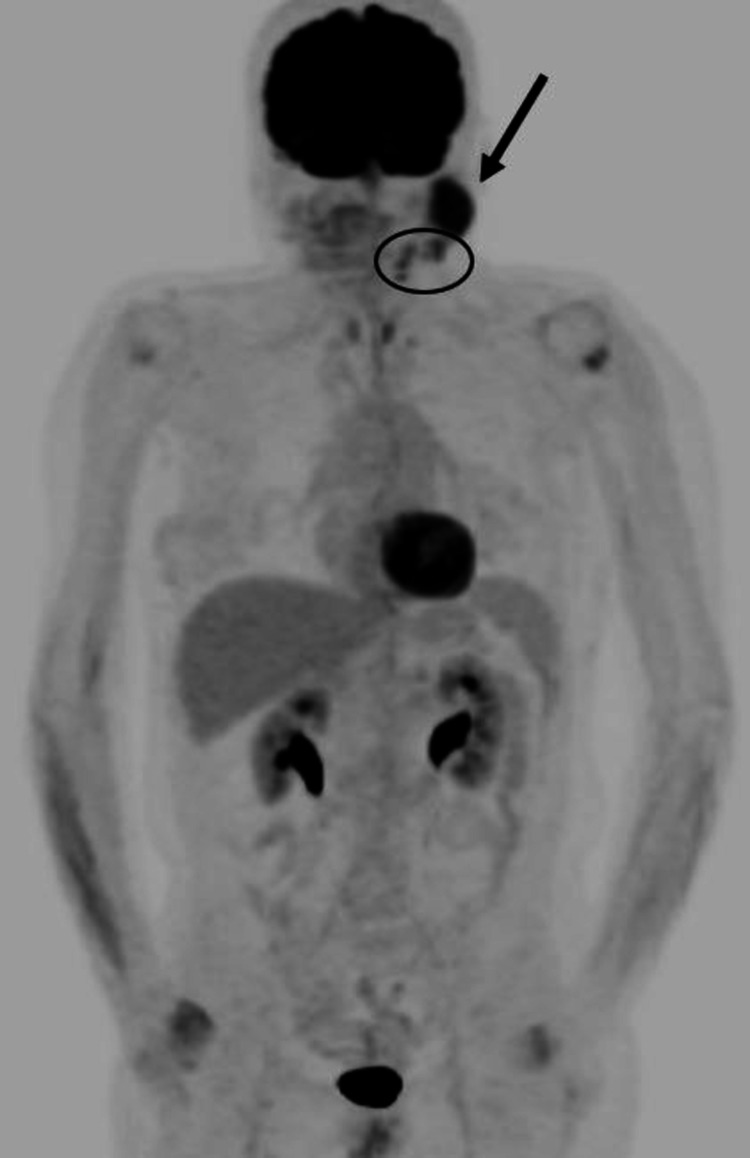
FDG-18 PET MIP image showing intense radiotracer uptake in left parotid space mass (black arrow). The additional sites of radiotracer uptake in left level II cervical lymph nodes (black circle) consistent with nodal involvement. The patient information at the time of injection: blood glucose - 84 mg/dL, weight - 122 lbs, BMI - 22 kg/m^2^, and time to injection - 64 minutes. FDG-18: fludeoxyglucose F-18; PET: positron emission tomography; MIP: maximum intensity projection

The patient was referred to otolaryngology where tissue sampling was performed which revealed primary MALT non-Hodgkin lymphoma of the left parotid gland (Figures 4, 5). Once the diagnosis was confirmed, the patient went on to have a whole body fludeoxyglucose (FDG) F-18 positron emission tomography (PET) scan which showed high metabolic activity of the primary parotid neoplasm as well as nodal involvement of level II cervical lymph nodes on the ipsilateral side (Figure 6). 

## Discussion

Due to the non-specific clinical presentation of parotid gland neoplasms, imaging plays a paramount role in their diagnosis and eventual treatment. Before proceeding to imaging, clinicians should take a detailed history and conduct a thorough physical examination to offer clues as to which imaging modality may be the most effective to use during the next step in the diagnosis. The details of the medical history, including elapsed time since first noticing the mass, growth of the mass, presence of pain or facial numbness, history of other head, neck, or skin cancers, and the presence of comorbidities (i.e., Sjogren syndrome) should all be elicited from the patient. Physical examination, detailing size, mobility, fixation of the mass to adjacent anatomic structures, local or distant lymphadenopathy, and findings suggestive of facial nerve paralysis can also provide important clues to help the clinician develop a potential diagnosis [9,10]. Although clinical findings can help recognize masses, imaging is the gold standard for diagnosis and guiding further management.

Imaging studies to include ultrasound (US), CT, or MRI can be performed to determine the size and location of the neoplasm. Often, the extent of tumor involvement is much more accurately appreciated on either CT or MRI than is suggested by physical examination [11,12]. The osseous findings such as erosion or expansion of the temporal bone or mandible are best appreciated on CT while soft tissue infiltration, intracranial extension, and perineural invasion are best detected with MRI. These are complementary examinations that should both be performed as they provide information critical for diagnosis and surgical planning. Evidence of metastasis can also be elicited which is important in the final staging of cancer [13]. Considering the superficial location of salivary glands, ultrasound imaging can also play a timely and cost-efficient role in the initial evaluation of a suspected salivary gland neoplasm [14]. While final pathology cannot be definitively ascertained without the analysis of a pathological tissue sample, certain imaging findings are reliable indicators of benign or malignant neoplasms. Ill-defined margins on a contrast-enhanced MRI have been shown as the best predictor of malignant neoplasm [15]. In our case, homogeneous enhancement with ill-defined margins, and restricted diffusion with very low ADC values offered indications for the specific diagnosis of lymphoma [16]. Once medical history, physical examination, and radiologic imaging have been considered, the final diagnosis can be made with tissue analysis, with a sample obtained through either fine-needle aspiration or ultrasound-guided core needle biopsy. 

The management of most parotid gland neoplasms involves surgical resection. Benign or low-grade malignancies can often be treated with surgery alone, while higher-grade malignancies require surgery with adjuvant chemotherapy and/or radiation therapy. The surgical approach is largely dictated by pre-surgical imaging, with efforts taken to remove all affected tissue, while attempting to spare the facial nerve [17]. While surgery plays a considerable role in the management of most parotid gland neoplasms, it is unnecessarily invasive in the treatment of parotid gland lymphoma. In general, parotid gland lymphoma treatment varies based on the specific histologic subtype. The studies have suggested that in low-stage MALT lymphoma of the parotid, chemotherapy alone results in similar outcomes to those of radiation therapy alone. However, in more aggressive subtypes such as diffuse large B-cell lymphoma, a combination of chemotherapy and radiation therapy is usually recommended [18]. Our case offers a clear example of a patient for whom radiological studies, considered in the context of her past medical history, and confirmed by pathologic analysis, avoided unnecessary surgery in the management of her parotid gland neoplasm.

## Conclusions

The primary MALT non-Hodgkin lymphoma of the parotid gland is a rare neoplasm though it is most commonly seen in the setting of Sjogren syndrome. When patients present with symptoms concerning a salivary gland neoplasm, imaging plays a critical role in the diagnosis. While US can be a good initial screening tool, CT and MRI are the primary modalities for characterization and to help drive further management. Certain findings can be reliable indicators for benign tumors which need no further workup or malignant entities which would need a biopsy to determine further treatment. This case shows a rare tumor with classic imaging features which led to appropriate diagnosis and subsequent management.
